# Nafamostat Mesylate Improved Survival Outcomes of Sepsis Patients Who Underwent Blood Purification: A Nationwide Registry Study in Japan

**DOI:** 10.3390/jcm9082629

**Published:** 2020-08-13

**Authors:** Hiroshi Kamijo, Katsunori Mochizuki, Yuta Nakamura, Kotaro Mori, Michitaro Ichikawa, Kenichi Nitta, Hiroshi Imamura

**Affiliations:** 1 Department of Emergency and Critical Care Medicine, Shinshu University School of Medicine, 3-1-1 Asahi, Matsumoto 390-8621, Japan; hkamijo@shinshu-u.ac.jp (H.K.); kmori@shinshu-u.ac.jp (K.M.); qqichikawa@shinshu-u.ac.jp (M.I.); nittaken@shinshu-u.ac.jp (K.N.); imamura@shinshu-u.ac.jp (H.I.); 2 Department of Emergency Medicine, Saiseikai Kumamoto Hospital, 5-3-1 Chikami Minami-ku, Kumamoto 861-4193, Japan; ghhhj309@yahoo.co.jp

**Keywords:** sepsis, blood purification, nafamostat mesylate, anticoagulant

## Abstract

Nafamostat mesylate (NM) is a synthetic serine protease inhibitor that can be used as an anticoagulant during blood purification in critically ill patients, as well as a treatment for disseminated intravascular coagulation. Although NM has been reported to reduce the risk of bleeding during blood purification, its effect on survival outcomes of patients who received blood purification treatments is unclear. We hypothesized that administration of NM during blood purification can reduce mortality in patients with sepsis. A post hoc analysis was conducted on a nationwide retrospective registry that included data from 3195 sepsis patients registered at 42 intensive care units throughout Japan. We evaluated the effect of NM on hospital mortality and bleeding complications using propensity score matching in 1216 sepsis patients who underwent blood purification in the intensive care unit (ICU). Two-hundred-and-sixty-eight pairs of propensity score-matched patients who received NM and conventional therapy were compared. Hospital and ICU mortality rates in the NM group were significantly lower than those in the conventional therapy group. However, rates of bleeding complications did not differ significantly between the two groups. These data suggest that administration of NM improved the survival outcomes of sepsis patients who underwent blood purification in the ICU.

## 1. Introduction

Sepsis is a life-threatening condition characterized by a systemic dysregulated immune response induced by infection, causing multiple organ dysfunction as well as high mortality [1]. Sepsis induces complicated systemic dysregulation, including abnormal hemodynamics, tissue hypoxia, altered red blood cell function, and microvascular dysfunction [2]. Therefore, research into effective treatments for sepsis is of global interest, and various interventions have been developed and investigated [3]. Development of the systemic inflammatory response occurs through mediators such as cytokines and/or endotoxins [4]. These mediators cause activation of blood cells, such as neutrophils and platelets, followed by vascular endothelial damage, vascular hyperpermeability, and finally multiple organ failure and septic shock. Blood purification, such as cytokine or endotoxin removal, has been attempted as an adjunctive therapy to control these mediators and the following host response [5].

Additionally, sepsis frequently complicates coagulopathy, known as sepsis-induced coagulopathy or disseminated intravascular coagulation (DIC), which worsens multiple organ dysfunction [6,7]. Further, coagulopathy and the inflammatory response adversely interact via crosstalk between their pathophysiological pathways [8]. Several anticoagulants have often been used in the treatment of sepsis to control coagulopathy [9,10,11]. Although blood purification and anticoagulants for DIC treatment may be administered simultaneously, continuous intracircuit infusion of an anticoagulant is generally administered during blood purification, such as low molecular weight heparin (LMWH), undifferentiated heparin, or nafamostat mesylate (NM) [12,13]. In addition to the increased bleeding risk due to anticoagulant therapy, DIC itself sometimes causes bleeding complications [14]. Therefore, clinicians must consider bleeding risks and benefits of anticoagulants for continuous intracircuit infusion when performing blood purification in patients with sepsis.

NM is a synthetic serine protease inhibitor that can be used as an anticoagulant for DIC treatments, and its clinical application in coronavirus disease 2019 treatment is also being attempted [15]. Due to its short half-life, NM was anticipated to reduce bleeding risks during blood purification in critically ill patients, and is widely used for intracircuit anticoagulation in Korea and Japan [16]. NM reportedly reduces the risk of bleeding during blood purification in critically ill patients [17], and is a safe and effective anticoagulant in patients undergoing blood purification at high risk of bleeding [18]. However, data on bleeding risks in sepsis patients is limited. Furthermore, the effects of NM on survival outcomes of sepsis patients who received blood purification treatment are unclear. Therefore, the purpose of the present study was to retrospectively compare the rates of bleeding complications and hospital mortality between NM and conventional therapy to investigate the effect of NM on survival outcomes of sepsis patients who underwent blood purification.

## 2. Materials and Methods

### 2.1. Study Design, Setting, and Population

This study was conducted as a post hoc analysis of a retrospective cohort dataset of consecutive adult patients admitted to 42 intensive care units (ICU) at 40 institutions throughout Japan and treated for severe sepsis or septic shock between January 2011 and December 2013 (the Japan Septic Disseminated Intravascular Coagulation (J-Septic DIC) registry) [19]. We evaluated the effect of NM on survival outcomes using propensity score matching in patients who underwent blood purification in the ICU.

**Figure 1:**
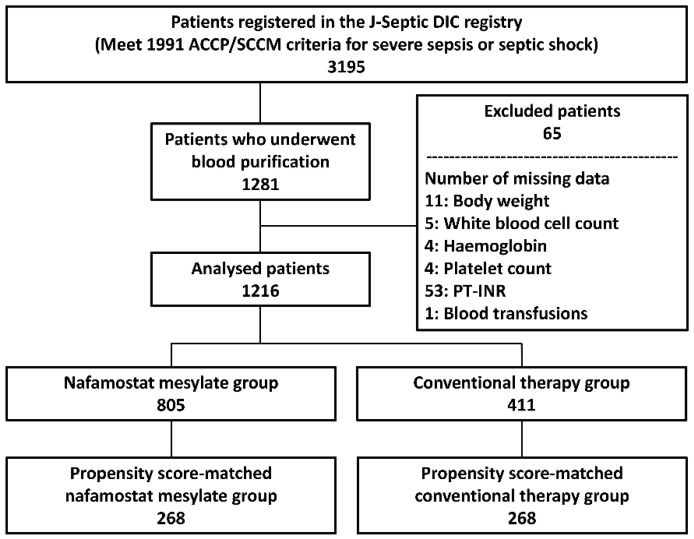
Study flowchart. Numbers of patients are indicated in each box. ACCP: American College of Chest Physicians; J-Septic DIC: Japan Septic Disseminated Intravascular Coagulation; PT-INR: Prothrombin time international normalized ratio; SCCM: Society of Critical Care Medicine.

Severe sepsis and septic shock were defined in the registry as per the conventional criteria proposed by the American College of Chest Physicians/Society of Critical Care Medicine consensus conference in 1991 [20]. Patients ≥ 18 years of age with severe sepsis or septic shock upon ICU admission were enrolled in the registry. We included sepsis patients who received any of following blood purification treatments in the ICU: renal replacement therapy (RRT) (renal and/or non-renal indication), polymyxin B-immobilized hemoperfusion (PMX-HP), and/or plasma exchange (PE). We excluded patients with missing data in any of the analyzed variables, such as body weight, laboratory data on day 1, and data related to treatments (Figure 1).

### 2.2. Analyzed Data

The present study analyzed variables collected in the J-Septic DIC registry after excluding variables with ≥10% missing data.

The following ICU and patient characteristics were analyzed: general or emergency ICU, management policy of the ICU, number of beds in the ICU, admission route to the ICU, age, sex, body weight, pre-existing organ dysfunction, and hemostatic disorder, APACHE II score on day 1, SOFA score on day 1, SIRS score on day 1, white blood cell count on day 1, level of hemoglobin on day 1, platelet count on day 1, prothrombin time-international normalized ratio on day 1, blood culture results, microorganisms responsible for sepsis, and primary infection site.

The following therapeutic variables were analyzed; surgical interventions at the infection site, use of mechanical ventilator, use of vasopressor, administration of immunoglobulins, administration of low doses of steroids, veno-arterial extracorporeal membrane oxygenation (ECMO), veno-venous ECMO, intra-aortic balloon pumping, administration of anticoagulant for DIC treatment during the first 7 days of ICU stay (antithrombin, thrombomodulin, protease inhibitors, and/or heparinoids), administration of anti-thrombotic drugs for other than DIC treatment during the first 7 days of ICU stay (NM, heparin, warfarin, anti-platelet drugs, and/or others), administration of blood transfusions during the first 7 days of ICU stay (red blood cell concentration, fresh frozen plasma, and/or platelet concentration), and blood purifications during the first 7 days of ICU stay (RRT, RRT for non-renal indications, PMX-HP, and/or PE).

The following numerical variables were analyzed; number of beds in the ICU, age, body weight, severity scores, laboratory data, and administration of blood transfusions. Other variables were analyzed as categorical variables.

### 2.3. Outcome Measures

The primary outcome of the study was hospital mortality. The secondary outcomes of the study were ICU mortality and bleeding complications during the first 7 days of ICU stay (bleeding received transfusion, intracranial hemorrhage, bleeding required therapeutic intervention, and/or bleeding to death).

### 2.4. Statistical Analysis

Propensity scores for administration of NM were estimated using a logistic regression model based on the covariates ICU and patient characteristics, as well as therapeutic variables that diminished administration of NM. The C statistic of the propensity score was estimated using receiver operating characteristic curve analysis. To control for background differences between the two groups, we implemented 1:1 nearest neighbor matching without replacement within a propensity score caliper width of 0.2 of the standard deviation of the logit of the propensity score. Propensity score-matched NM and non-NM (conventional therapy) groups were compared in terms of their outcome variables. Categorical variables were compared using the chi-squared test. Kaplan–Meier survival curves of the two groups were plotted with interval-censored data, and survival times were compared between the two groups using log-rank tests.

Categorical variables were presented as numbers and percentages, whereas numerical variables were summarized using medians (interquartile range). We did not impute any missing data and performed complete case analysis for all analyses. Standardized differences were calculated using R version 3.6.2, and all other statistical analyses were performed using IBM SPSS Statistics version 26 (IBM Corp., Armonk, NY, USA). Differences were considered statistically significant if *p* < 0.05.

## 3. Results

### 3.1. ICU and Patient Characteristics

Among the 3195 patients who were registered to the J-Septic DIC registry, we included 1216 patients who underwent blood purification in the final cohort (*n* = 805, NM group; *n* = 411, conventional therapy group) (Figure 1). The ICU and patient characteristics of the final cohort are shown in Table 1 and Table 2. Hospital and ICU mortalities of the cohort were 41.2% (501/1216) and 26.4% (321/1216), respectively. The rate of bleeding complications during the first 7 days of ICU stay of the cohort was 16.3% (198/1216).

Propensity scores were calculated for administration of NM, which revealed a C statistic of 0.855, a Hosmer–Lemeshow chi-squared value of 10.259 (df = 8), and a *p*-value of 0.247. Propensity score matching created 268 pairs with appropriate balance, after which the standardized differences of all patient background variables were <0.1 [21]

### 3.2. Comparing Propensity Score-Matched Nafamostat Mesylate and Conventional Therapy Groups

Table 3 shows patient outcomes in the NM and conventional therapy groups. In the propensity score-matched cohort, patients in the NM group had significantly lower hospital and ICU mortalities than those in the conventional therapy group (29.5% (79/268) vs. 40.3% (108/268), *p* = 0.009; 18.3% (49/268) vs. 26.9% (72/268), *p* = 0.017, respectively). Rates of bleeding complications during the first 7 days of ICU stay did not differ significantly between the two groups.

**Figure 2:**
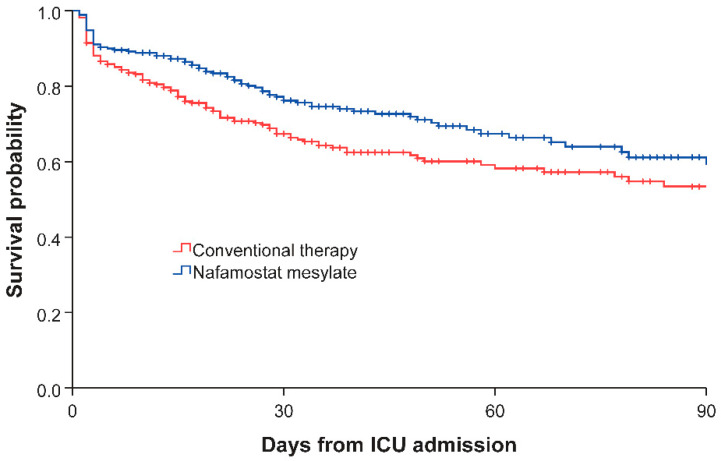
Survival curves of the nafamostat mesylate and conventional therapy groups. ICU: intensive care unit.

Kaplan-Meier survival curves of the NM and conventional therapy groups are illustrated in Figure 2. The NM group demonstrated a significantly longer survival time than the conventional therapy group (log-rank test; *p* = 0.011).

## 4. Discussion

In this post hoc analysis of the Japanese nationwide registry, NM significantly reduced hospital and ICU mortalities in sepsis patients who received blood purification treatment compared with conventional therapy. However, bleeding complications did not differ significantly between the two groups. The present study is the first to demonstrate that administration of NM significantly improved survival outcomes in patients with sepsis in the ICU.

Choi et al. reported that, compared with no anticoagulant (NA), NM improved survival outcomes without increasing bleeding risk during continuous renal replacement therapy (CRRT) in critically ill patients with acute kidney injury and high risk of bleeding. Although the results were not statistically significant due to their small sample size (55 subjects), lower hospital and 90-day mortalities were observed in the NM group compared with the NA group (47.2% vs. 52.8%, *p* =  0.054; 57.8% vs. 88.8%, *p*  =  0.063, respectively) in this randomized controlled trial (RCT) [22]. The authors suggested that the improved outcome might be caused by NM extending the hemofilter lifespan, which resulted in effective fluid removal and clearance in the NM group. Further, they suggested that a study with a larger sample size might have proven statistically significant. The present study with a larger sample size (268 pairs) supports that NM positively impacts survival outcomes. However, the results of an RCT reported by Lee et al. [18] contrarily suggested similar mortalities in the NM and NA groups. Therefore, the reduced mortality observed in the current study may the result of a different mechanism from that suggested by Choi et al. [22] Further, both groups in the study by Lee et al. [18] exhibited extremely high hospital mortalities (NM 75.0%, NA 74.1%), which differs from that reported for the conventional therapy group in the present study (40.3%). Considering this difference, the expected NM benefit to survival outcome may not be observed in a population with high mortality. Additional study is warranted to identify specific population criteria to optimize the benefits of NM therapy.

Whereas the above-mentioned RCTs [18,22] employed NA as controls, specific anticoagulant type was not recorded in the dataset used for the conventional therapy group in the present study. Arimura et al. reported the anticoagulant use rate for blood purification among critically ill patients in Japan for 2013, which corresponds with the timing of the present study. According to this report, NM, unfractionated heparin (UFH), LMWH, and others were used 84.3%, 11.5%, 2.4%, and 1.8% in 2013 for blood purification in critically ill Japanese patients, respectively [13]. Although this report did not contain all the anticoagulants used in the present study, the conventional therapy group in the present study most likely included mainly UFH or LMWH within this time period. In fact, a previous worldwide study revealed that UFH was the most common choice among critically ill patients who received anticoagulation treatment during blood purification [12]. However, Yatabe et al. suggested in their systematic review of anticoagulant treatments for patients with sepsis-induced DIC that heparin should be used with caution due to risk of bleeding [9], and heparin combined with antithrombin is known to increase bleeding complications [23]. Therefore, using heparin during blood purification on patients undergoing anticoagulant therapy for sepsis-induced DIC may increase the risk of bleeding complications. According to a systematic review reported by Kang et al., NM administration may induce less bleeding risk than heparin during CRRT [24]. Further, NM has potent pharmacological antifibrinolytic activity, in addition to its ability to inhibit blood coagulation factors [25]. Although sepsis-induced DIC is typically classified as suppressed-fibrinolytic-type [26], both coagulation and fibrinolytic systems are activated, and Suzuki et al. reported the bleeding type comprised more than 40% of sepsis-induced DIC cases [14]. Many cases in the current cohort received anticoagulant therapy for DIC or other than DIC, thus NM may be optimally used as an additional anticoagulant to avoid elevated bleeding risks induced by progression of DIC. Although bleeding complications did not differ significantly between the NM and conventional therapy groups in the current study, this may be because bleeding complications were only recorded for 7 days after ICU admission. Therefore, prospective studies that include long-term observation of bleeding complications should determine the impact of NM on bleeding risk and its relationship with survival outcome.

The use of a dataset with a large number of cases in which blood purification was performed using NM is a particular strength of the present study. Even after propensity score matching, the present study comprised a larger number of patients (268) who received NM for blood purification than the above-mentioned systematic review by Kang et al. that included not only RCT but also non-RCT patients (252). Although observing significant reductions of mortality in studies examining anticoagulants for blood purification is difficult [27], the relatively large sample size of this dataset enabled us to evaluate the significance of NM administration on survival outcomes. Further, we recently reported that the dataset used in the present study includes a significant positive effect modification on survival outcomes of sepsis between thrombomodulin-alpha and PMX-HP treatment [28]. The bias-reducing capabilities of propensity scores may be decreased if propensity scores are estimated without considering interactions [29], therefore we controlled for effect modification when estimating propensity scores in the present study. To the best of our knowledge, this is the first analysis of this dataset to consider effect modification.

On the other hand, there are several limitations in this study. First, this study is a retrospective study, and the design may be associated with risk of unmeasured or unknown biases. Second, the dataset used in the present study was compiled prior to 2016 when the current definition of sepsis was adopted [30]. Therefore, severe sepsis as defined in this dataset differs from the current definition. Third, this dataset does not provide detailed information regarding blood purification treatments, such as hemofilter material for renal replacement therapy or NM dose. Especially, hemofilters using acrylonitrile and methallyl sulfonate copolymer (AN69-ST) membranes are known to adsorb NM [31,32], therefore the beneficial effect of NM observed in the present study may have been influenced in cases using the AN69-ST hemofilter. However, this limitation is unlikely because the AN69-ST hemofilter was not approved for CRRT use in Japan during the period of the current study. Fourth, although cardiovascular dysfunction is a common and important complication in sepsis [33], the dataset used in the present study did not include detailed cardiovascular parameters; thus, we could not evaluate the response to resuscitation or outcomes based on them. Further studies are warranted to validate our findings; however, the results of the present study will help design future RCTs to evaluate the effects of NM and impact clinical decision-making.

## 5. Conclusions

In a post hoc analysis of the Japanese nationwide retrospective registry, administration of NM significantly reduced hospital and ICU mortalities in sepsis patients who underwent blood purification compared with conventional anticoagulant therapy. Further prospective research, including detailed studies regarding blood purification treatments and long-term observation of bleeding complications as well as cardiovascular parameters, is warranted to optimize the benefits of NM therapy, evaluate its impact on bleeding risk, and further investigate the effects of blood purification with NM for septic patients.

## Figures and Tables

**Table 1:** Patient characteristics and treatments in the nafamostat mesylate and conventional therapy groups.

	Overall (*n* = 1216)	Nafamostat Mesylate (*n* = 805)	Conventional Therapy (*n* = 411)	Standardized Difference
**Characteristics of ICU**				0.113
General ICU, *n* (%)	698 (57.4)	447 (55.5)	251 (61.1)	
Emergency ICU, *n* (%)	518 (42.6)	358 (44.5)	160 (38.9)	
**Management Policy of the ICU**				0.474
Closed Policy, *n* (%)	605 (49.8)	447 (55.5)	158 (38.4)	
Open Policy, *n* (%)	381 (31.3)	255 (31.7)	126 (30.7)	
Other, *n* (%)	230 (18.9)	103 (12.8)	127 (30.9)	
**Number of Beds in the ICU**	12 (8, 16)	12 (8, 14)	11 (7, 18)	0.045
**Admission Route to the ICU**				0.067
Emergency Department, *n* (%)	465 (38.2)	299 (37.1)	166 (40.4)	
Other Hospital, *n* (%)	315 (25.9)	213 (26.5)	102 (24.8)	
Ward, *n* (%)	436 (35.9)	293 (36.4)	143 (34.8)	
**Age, (Years)**	70 (61, 78)	70 (62, 78)	70 (60, 79)	0.066
**Male Sex, *n* (%)**	750 (61.7)	510 (63.4)	240 (58.4)	0.102
**Body Weight, (Kg)**	56.1 (48.0, 65.4)	56.3 (48.0, 66.0)	56.1 (48.0, 65.0)	0.064
**Pre-Existing Organ Insufficiency or Immunosuppression Based on APACHE II Score**				
Liver, *n* (%)	69 (5.7)	47 (5.8)	22 (5.4)	0.021
Respiratory, *n* (%)	33 (2.7)	19 (2.4)	14 (3.4)	0.063
Cardiovascular, *n* (%)	76 (6.3)	52 (6.5)	24 (5.8)	0.026
Renal, *n* (%)	194 (16.0)	141 (17.5)	53 (12.9)	0.129
Immunocompromised, *n* (%)	209 (17.2)	138 (17.1)	71 (17.3)	0.003
**Pre-Existing Hemostatic Disorder**				
Cirrhosis, *n* (%)	60 (4.9)	39 (4.8)	21 (5.1)	0.012
Hematologic Malignancy, *n* (%)	40 (3.3)	29 (3.6)	11 (2.7)	0.053
Chemotherapy, *n* (%)	63 (5.2)	44 (5.5)	19 (4.6)	0.039
Warfarin Intake, *n* (%)	68 (5.6)	40 (5.0)	28 (6.8)	0.078
Other, *n* (%)	31 (2.5)	15 (1.9)	16 (3.9)	0.122
**APACHE II Score**	25 (19, 31)	27 (21, 32)	22 (17, 28)	0.352
**SOFA Score**	12 (9, 14)	12 (9, 14)	11 (8, 13)	0.318
**SIRS Score**	3 (2, 4)	3 (2, 4)	3 (3, 4)	0.115
**White Blood Cell Count, ×10^3^**	10.3 (3.5, 17.7)	10.1 (3.6, 17.1)	11.3 (3.4, 19.2)	0.072
**Hemoglobin, G/Dl**	10.3 (8.7, 12.2)	10.4 (8.7, 12.2)	10.3 (8.7, 12.2)	0.032
**Platelet Count, ×10^3^**	103 (54, 170)	100 (53, 165)	111 (59, 178)	0.110
**Prothrombin Time International Normalized Ratio**	1.42 (1.22, 1.76)	1.41 (1.22, 1.76)	1.42 (1.22, 1.73)	0.027
**Blood Culture**				0.141
Not Taken, *n* (%)	61 (5.0)	34 (4.2)	27 (6.6)	
Positive, *n* (%)	558 (45.9)	385 (47.8)	173 (42.1)	
Negative, *n* (%)	597 (49.1)	386 (48.0)	211 (51.3)	
**Microorganisms**				0.168
Unknown, *n* (%)	267 (22.0)	169 (21.0)	98 (23.8)	
Virus, *n* (%)	8 (0.7)	6 (0.7)	2 (0.5)	
Gram-Negative Rod, *n* (%)	436 (35.9)	291 (36.1)	145 (35.3)	
Gram-Positive Coccus, *n* (%)	286 (23.5)	202 (25.1)	84 (20.4)	
Fungus, *n* (%)	18 (1.5)	12 (1.5)	6 (1.5)	
Mixed Infection, *n* (%)	183 (15.0)	117 (14.5)	66 (16.1)	
Other, *n* (%)	18 (1.5)	8 (1.0)	10 (2.4)	
**Primary Source of Infection**				0.218
Unknown, *n* (%)	87 (7.2)	55 (6.8)	32 (7.8)	
Catheter-Related Bloodstream Infection, *n* (%)	17 (1.4)	13 (1.6)	4 (1.0)	
Bone, Soft Tissue, *n* (%)	138 (11.3)	97 (12.0)	41 (10.0)	
Cardiovascular System, *n* (%)	29 (2.4)	22 (2.7)	7 (1.7)	
Central Nervous System, *n* (%)	15 (1.2)	14 (1.7)	1 (0.2)	
Urinary Tract, *n* (%)	138 (11.3)	94 (11.7)	44 (10.7)	
Lung, Thoracic Cavity, *n* (%)	239 (19.7)	161 (20.0)	78 (19.0)	
Abdomen, *n* (%)	530 (43.6)	333 (41.4)	197 (47.9)	
Other, *n* (%)	23 (1.9)	16 (2.0)	7 (1.7)	
**Specific Treatment**				
Surgical Intervention, *n* (%)	640 (52.6)	410 (50.9)	230 (56.0)	0.101
Mechanical Ventilator, *n* (%)	1038 (85.4)	696 (86.5)	342 (83.2)	0.091
Vasopressor, *n* (%)	1109 (91.2)	743 (92.3)	366 (89.1)	0.112
Immunoglobulins, *n* (%)	505 (41.5)	376 (46.7)	129 (31.4)	0.318
Low-Dose Steroids, *n* (%)	447 (36.8)	315 (39.1)	132 (32.1)	0.147
Veno-Arterial ECMO, *n* (%)	17 (1.4)	9 (1.1)	8 (1.9)	0.067
Veno-Venous ECMO, *n* (%)	30 (2.5)	21 (2.6)	9 (2.2)	0.027
Intra-Aortic Balloon Pumping, *n* (%)	9 (0.7)	4 (0.5)	5 (1.2)	0.078
Red Blood Cell Transfusion, Units	3 (0, 6)	4 (0, 6)	2 (0, 6)	0.167
Fresh Frozen Plasma Transfusion, Units	0 (0, 10)	0 (0, 10)	0 (0, 8)	0.073
Platelet Concentration Transfusion, Units	0 (0, 20)	0 (0, 20)	0 (0, 15)	0.193
**Therapeutic Interventions For DIC**				
Antithrombin, *n* (%)	560 (46.1)	386 (48.0)	174 (42.3)	0.113
Thrombomodulin Alpha, *n* (%)	484 (39.8)	361 (44.8)	123 (29.9)	0.312
Protease Inhibitors, *n* (%)	214 (17.6)	125 (15.5)	89 (21.7)	0.158
Heparinoids, *n* (%)	70 (5.8)	48 (6.0)	22 (5.4)	0.026
**Anti-Thrombotic Drugs for Other Than DIC**				
Heparin, *n* (%)	175 (14.4)	74 (9.2)	101 (14.4)	0.420
Warfarin, *n* (%)	13 (1.1)	6 (0.7)	7 (1.7)	0.087
Anti-Platelet Drugs, *n* (%)	26 (2.1)	4 (0.5)	22 (5.4)	0.291
Other, *n* (%)	8 (0.7)	4 (0.5)	4 (1.0)	0.056
**Blood Purification Treatments**				
RRT, *n* (%)	842 (69.2)	640 (79.5)	202 (49.1)	0.668
RRT For Non-Renal Indications, *n* (%)	254 (20.9)	180 (22.4)	74 (18.0)	0.109
PMX-HP, *n* (%)	663 (54.5)	425 (52.8)	238 (57.9)	0.103
Plasma Exchange, *n* (%)	28 (2.3)	15 (1.9)	13 (3.2)	0.083
**Concomitant Treatment with Thrombomodulin Alpha And PMX-HP, *n* (%)**	285 (23.4)	216 (26.8)	69 (16.8)	0.245

Data are presented as *n* (%), or median (interquartile range). APACHE: acute physiology and chronic health evaluation; DIC: disseminated intravascular coagulation; ECMO: extracorporeal membrane oxygenation; ICU: intensive care unit; PMX-HP: polymyxin B-immobilized hemoperfusion; RRT: renal replacement therapy; SIRS: systemic inflammatory response syndrome; SOFA: sequential organ failure assessment.

**Table 2:** Patient characteristics and treatments in the nafamostat mesylate and conventional therapy groups after propensity score matching.

	Nafamostat Mesylate (*n* = 268)	Conventional Therapy (*n* = 268)	Standardized Difference
**Characteristics of the ICU**			0.008
General ICU, *n* (%)	166 (61.9)	165 (61.6)	
Emergency ICU, *n* (%)	102 (38.1)	103 (38.4)	
**Management Policy of the ICU**			0.037
Closed Policy, *n* (%)	116 (43.3)	115 (42.9)	
Open Policy, *n* (%)	90 (33.6)	87 (32.5)	
Other, *n* (%)	62 (23.1)	66 (24.6)	
**Number of Beds in the ICU**	12 (8, 16)	12 (7, 18)	0.068
**Admission Route to the ICU**			0.083
Emergency Department, *n* (%)	120 (44.8)	109 (40.7)	
Other Hospital, *n* (%)	64 (23.9)	68 (25.4)	
Ward, *n* (%)	84 (31.3)	91 (34.0)	
**Age, (Years)**	70 (61, 78)	71 (61, 78)	0.003
**Male Sex, *n* (%)**	174 (64.9)	165 (61.6)	0.070
**Body Weight, (Kg)**	57.6 (49.8, 67.0)	55.5 (47.9, 65.0)	0.070
**Pre-Existing Organ Insufficiency or Immunosuppression Based on APACHE II Score**			
Liver, *n* (%)	15 (5.6)	12 (4.5)	0.051
Respiratory, *n* (%)	8 (3.0)	8 (3.0)	0
Cardiovascular, *n* (%)	14 (5.2)	16 (6.0)	0.032
Renal, *n* (%)	38 (14.2)	39 (14.6)	0.011
Immunocompromised, *n* (%)	36 (13.4)	38 (14.2)	0.022
**Pre-Existing Hemostatic Disorder**			
Cirrhosis, *n* (%)	14 (5.2)	9 (3.4)	0.092
Hematologic Malignancy, *n* (%)	7 (2.6)	7 (2.6)	0
Chemotherapy, *n* (%)	13 (4.9)	12 (4.5)	0.018
Warfarin Intake, *n* (%)	12 (4.5)	13 (4.9)	0.018
Other, *n* (%)	6 (2.2)	7 (2.6)	0.024
**APACHE II Score**	25 (18, 30)	23 (17, 29)	0.015
**SOFA Score**	11 (8, 13)	11 (8, 13)	0.011
**SIRS Score**	3 (2, 4)	3 (3, 4)	0.083
**White Blood Cell Count, ×10^3^**	11.2 (3.4, 18.2)	12.0 (4.5, 19.5)	0.090
**Hemoglobin, G/Dl**	10.6 (8.9, 12.6)	10.5 (8.8, 12.5)	0.002
**Platelet Count, ×10^3^**	118 (65, 175)	109 (57, 186)	0.009
**Prothrombin Time International Normalize Ratio**	1.40 (1.21, 1.73)	1.38 (1.20, 1.70)	0.013
**Blood Culture**			0.023
Not Taken, *n* (%)	14 (5.2)	14 (5.2)	
Positive, *n* (%)	114 (42.5)	117 (43.7)	
Negative, *n* (%)	140 (52.2)	137 (51.1)	
**Microorganisms**			0.090
Unknown, *n* (%)	66 (24.6)	62 (23.1)	
Virus, *n* (%)	0 (0)	0 (0)	
Gram-Negative Rod, *n* (%)	106 (39.6)	101 (37.7)	
Gram-Positive Coccus, *n* (%)	56 (20.9)	57 (21.3)	
Fungus, *n* (%)	3 (1.1)	4 (1.5)	
Mixed Infection, *n* (%)	32 (11.9)	39 (14.6)	
Other, *n* (%)	5 (1.9)	5 (1.9)	
**Primary Source of Infection**			0.087
Unknown, *n* (%)	24 (9.0)	24 (9.0)	
Catheter-Related Bloodstream Infection, *n* (%)	2 (0.7)	2 (0.7)	
Bone, Soft Tissue, *n* (%)	29 (10.8)	28 (10.4)	
Cardiovascular System, *n* (%)	4 (1.5)	6 (2.2)	
Central Nervous System, *n* (%)	1 (0.4)	1 (0.4)	
Urinary Tract, *n* (%)	31 (11.6)	26 (9.7)	
Lung, Thoracic Cavity, *n* (%)	46 (17.2)	50 (18.7)	
Abdomen, *n* (%)	126 (47.0)	126 (47.0)	
Other, *n* (%)	5 (1.9)	5 (1.9)	
**Specific Treatment**			
Surgical Intervention, *n* (%)	146 (54.5)	151 (56.3)	0.038
Mechanical Ventilator, *n* (%)	224 (83.6)	221 (82.5)	0.030
Vasopressor, *n* (%)	240 (89.6)	247 (92.2)	0.091
Immunoglobulins, *n* (%)	97 (36.2)	96 (35.8)	0.008
Low-Dose Steroids, *n* (%)	84 (31.3)	86 (32.1)	0.016
Veno-Arterial ECMO, *n* (%)	3 (1.1)	3 (1.1)	0
Veno-Venous ECMO, *n* (%)	5 (1.9)	6 (2.2)	0.026
Intra-Aortic Balloon Pumping, *n* (%)	2 (0.7)	1 (0.4)	0.050
Red Blood Cell Transfusion, Units	2 (0, 6)	2 (0, 6)	0.008
Fresh Frozen Plasma Transfusion, Units	0 (0, 10)	0 (0, 8)	0.014
Platelet Concentration Transfusion, Units	0 (0, 10)	0 (0, 15)	0.053
**Therapeutic Interventions For DIC**			
Antithrombin, *n* (%)	119 (44.4)	116 (43.3)	0.023
Thrombomodulin Alpha, *n* (%)	99 (36.9)	90 (33.6)	0.070
Protease Inhibitors, *n* (%)	45 (16.8)	49 (18.3)	0.039
Heparinoids, *n* (%)	17 (6.3)	14 (5.2)	0.048
**Anti-Thrombotic Drugs for Other Than DIC**			
Heparin, *n* (%)	51 (19.0)	55 (20.5)	0.037
Warfarin, *n* (%)	2 (0.7)	3 (1.1)	0.039
Anti-Platelet Drugs, *n* (%)	4 (1.5)	3 (1.1)	0.033
Other, *n* (%)	4 (1.5)	2 (0.7)	0.071
**Blood Purification Treatments**			
RRT, *n* (%)	154 (57.5)	149 (55.6)	0.038
RRT For Non-Renal Indications, *n* (%)	54 (20.1)	54 (20.1)	0
PMX-HP, *n* (%)	143 (53.4)	152 (56.7)	0.068
Plasma Exchange, *n* (%)	8 (3.0)	5 (1.9)	0.073
**Concomitant Treatment with Thrombomodulin Alpha And PMX-HP, *n* (%)**	59 (22.0)	55 (20.5)	0.036

Data are presented as *n* (%) or median (interquartile range). APACHE: acute physiology and chronic health evaluation; DIC: disseminated intravascular coagulation; ECMO: extracorporeal membrane oxygenation; ICU: intensive care unit; PMX-HP: polymyxin B-immobilized hemoperfusion; RRT: renal replacement therapy; SIRS: systemic inflammatory response syndrome; SOFA: sequential organ failure assessment.

**Table 3:** Outcomes in the nafamostat mesylate and conventional therapy groups.

	Unmatched			Matched		
	Nafamostat Mesylate (*n* = 805)	Conventional Therapy (*n* = 411)	*p*-Value	Nafamostat Mesylate (*n* = 268)	Conventional Therapy (*n* = 268)	*p*-Value
**Hospital Mortality, *n* (%)**	343 (42.6)	158 (38.4)	0.163	79 (29.5)	108 (40.3)	0.009
**ICU Mortality, *n* (%)**	226 (28.1)	95 (23.1)	0.063	49 (18.3)	72 (26.9)	0.017
**Bleeding Complications, *n* (%)**	129 (16.0)	69 (16.8)	0.733	43 (16.0)	50 (18.7)	0.425

Data are presented as *n* (%), or median (interquartile range).
